# Ascitic microbiota alteration is associated with portal vein tumor thrombosis occurrence and prognosis in hepatocellular carcinoma

**DOI:** 10.1128/mbio.00245-24

**Published:** 2024-04-02

**Authors:** Yingyun Guo, Shan Tian, Na Zhan, Chuan Liu, Jiao Li, Jiaming Hu, Meiqi Qiu, Binglu Huang, Weiguo Dong

**Affiliations:** 1 Department of Gastroenterology, Renmin Hospital of Wuhan University, Wuhan, Hubei, China; 2 Department of Infectious Disease, Union Hospital, Tongji Medical College, Huazhong University of Science and Technology, Wuhan, Hubei, China; 3 Department of Pathology, Renmin Hospital of Wuhan University, Wuhan, Hubei, China; 4 Department of Pathology, Union Hospital, Tongji Medical College, Huazhong University of Science and Technology, Wuhan, Hubei, China; College of Veterinary Medicine, Cornell university, Ithaca, New York, USA

**Keywords:** portal vein tumor thrombosis, malignant ascites, hepatocellular carcinoma, microbiome

## Abstract

Portal vein tumor thrombosis (PVTT) frequently leads to malignant ascites (MA) in individuals with hepatocellular carcinoma (HCC), remaining a bottleneck in the treatment. This study aimed to explore the differences in microbes in paired groups and provide novel insights into PVTT and MA-related treatments. Formalin-fixed paraffin embedding ascite samples were collected from MA secondary to HCC and benign ascites (BA) secondary to liver cirrhosis (LC). Ascitic microbiota profiles were determined in the HCC and LC groups by 16S rRNA sequencing. Prognostic risk factors were screened using survival analysis. The correlation between the significantly different microbial signatures in the groups with PVTT (WVT) and non-PVTT (NVT) and clinical characteristics was explored. The expression of different immune cells was determined by labeling four markers in the MA tissue chips using multiplex immunohistochemistry. A total of 240 patients (196 with HCC with MA and 44 with LC with BA) were included in this study. Microbial profiles differed between the HCC and LC groups. PVTT and Barcelona Clinic Liver Cancer stage were shown to be prognostic risk factors. Significant differences in the alpha and beta diversities were observed between the WVT and NVT groups. *Gammaproteobacteria* and *Acinetobacter* were the most abundant in the HCC MA. Differences in microbial signatures between the WVT and NVT groups were correlated with the level of C-reactive protein and apolipoprotein A1. This study revealed the microbial differences in the tumor microenvironment of MA secondary to HCC and BA secondary to LC.

**IMPORTANCE:**

First, we explored the alteration of the ascites ecosystem through the microbiota in patients with hepatocellular carcinoma (HCC) and liver cirrhosis. Second, this is the first clinical study to investigate the differences between patients with HCC with and without portal vein tumor thrombosis via 16S rRNA sequencing. These results revealed a decreased microbial diversity and metabolic dysregulation in individuals with HCC and portal vein tumor thrombosis. *Gammaproteobacteria* and *Acinetobacter* were the most abundant in the HCC malignant ascitic fluid. Our study provides a new perspective on treating malignant ascites secondary to HCC.

## INTRODUCTION

Hepatocellular carcinoma (HCC) is one of the most common malignancies and represents a major global healthcare challenge (1). Although significant progress has been made, there is room for improvement, particularly in developing systematic therapies (2). Malignant ascites, a serious complication of advanced HCC that results from peritoneal metastasis, has always been a significant clinical challenge in HCC management (3). Previous studies reported that portal vein tumor thrombosis (PVTT) frequently leads to ascites, portal vein hypertension, and tumor metastasis (4). As PVTT is detected in approximately 10%–40% of patients with HCC (5) and a sign of poor prognosis (6), it is important to seek new strategies for PVTT and malignant ascites arising from HCC.

With the rapid progression of sequencing technologies, 16S rRNA is effective in characterizing the microbiome features and compositional differences between subjects. Emerging evidence indicates a strong association between gut dysbacteriosis and HCC (7). Schneider et al. demonstrated that imbalanced gut microbiota may accelerate HCC development by shaping the hepatic inflammatory microenvironment (8). Chen et al. reported that the microbial composition of ascites in patients with cirrhosis is associated with short-term clinical outcomes (9). However, there has been no relevant research on the microbiota of malignant ascites secondary to HCC. Thus, gaining deeper insight into the changes in malignant ascites microbiota could provide a new perspective for treating refractory malignant ascites.

Herein, we investigated microbiota alterations in patients with malignant ascites secondary to HCC. We also explored the ascites microbial alterations and biochemical index changes in patients with or without PVTT. Finally, the correlation between PVTT status and immune cells in malignant ascites was investigated. This study aimed to better understand the interplay between ascitic microbiome and PVTT of ascites secondary to HCC and propose potential future directions.

## RESULTS

### Description of baseline features of included individuals with HCC and LC

**Fig 1:**
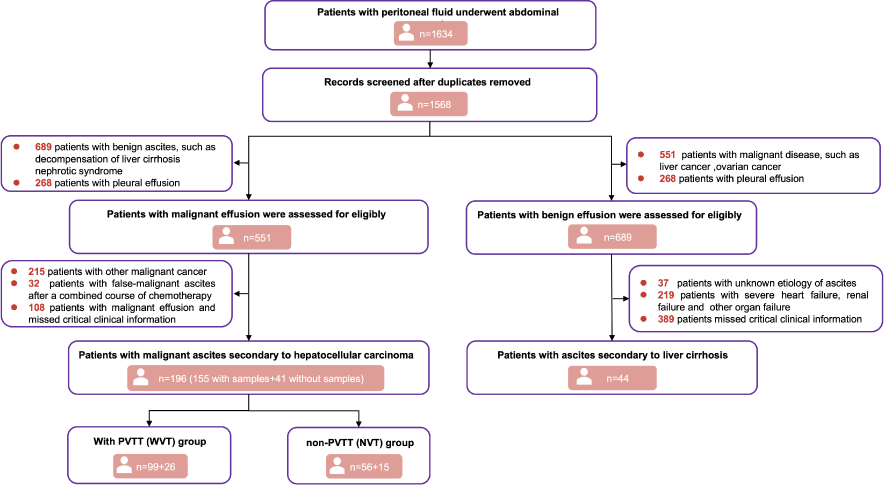
Flow diagram for patient inclusion and comparison cohort.

Figure 1 shows a flow diagram of the patient cohort. Table 1 shows the clinical details of the 240 patients who met the inclusion and exclusion criteria. In the HCC group (*n* = 196, 155 individuals with samples and 41 without samples), the median age of the HCC group was 61 (14–96), and 141 patients (71.9%) were male. HBV infection was observed in 128 patients (65.3%). Tumor differentiation was poor in 62 (31.6%), medium in 91 (46.4%), or well in 43 (21.9%) patients. The number of patients with Barcelona Clinic Liver Cancer (BCLC) stages A–B and C–D was 17 (8.7%) and 179 (91.3%), respectively. In the liver cirrhosis (LC) group (*n* = 44), the median age of the HCC group was 62 (32–82), and 27 patients (61.4%) were male. There were no apparent differences between the two groups in terms of age, sex, hepatitis B virus (HBV) infection rate, or smoking status.

**TABLE 1:** Clinical characteristics of HCC and LC patients^*a*^

Characteristics	HCC (*n* = 196)	LC (*n* = 44)	*P*
Age (year)	61 (14–96)	62 (32–82)	0.841
Gender (male)	141 (71.9%)	27 (61.4%)	0.167
Smoking status (never)	131 (66.8%)	35 (79.5%)	0.099
HBV infection (yes)	128 (65.3%)	28 (63.6%)	0.834
Tumor number (multiple)	169 (86.2%)	–	
Tumor differentiation		–	
Poor	62 (31.6%)	–	
Medium	91 (46.4%)	–	
Well	43 (21.9%)	–	
Tumor size (cm)		–	
<5	38 (19.4%)	–	
5–10	94 (48.0%)	–	
>10	64 (32.6%)	–	
BCLC stage		–	
A–B	17 (8.7%)	–	
C–D	179 (91.3%)	–	

^*a*^ En dash indicates these clinical characteristics do not apply to the liver cirrhosis group.

### Different microbial profiles between HCC and LC ascites samples

First, 16S rRNA sequencing was performed to determine the ascitic microbiota based on formalin-fixed paraffin-embedded (FFPE) tissues. A total of 155 FPPE tissues from malignant ascites samples secondary to HCC and 44 FPPE tissues from benign ascites samples secondary to LC were sequenced.

The rarefaction plot represents the HCC and LC groups, indicating a reliable sequencing depth for this analysis (Fig. S1A). This analysis identified 18,489 amplicon sequence variants (ASVs) in all samples. Among them, 9,896 and 6,453 ASVs were specific to samples from the HCC and LC groups, respectively (Fig. S1B).

**Fig 2:**
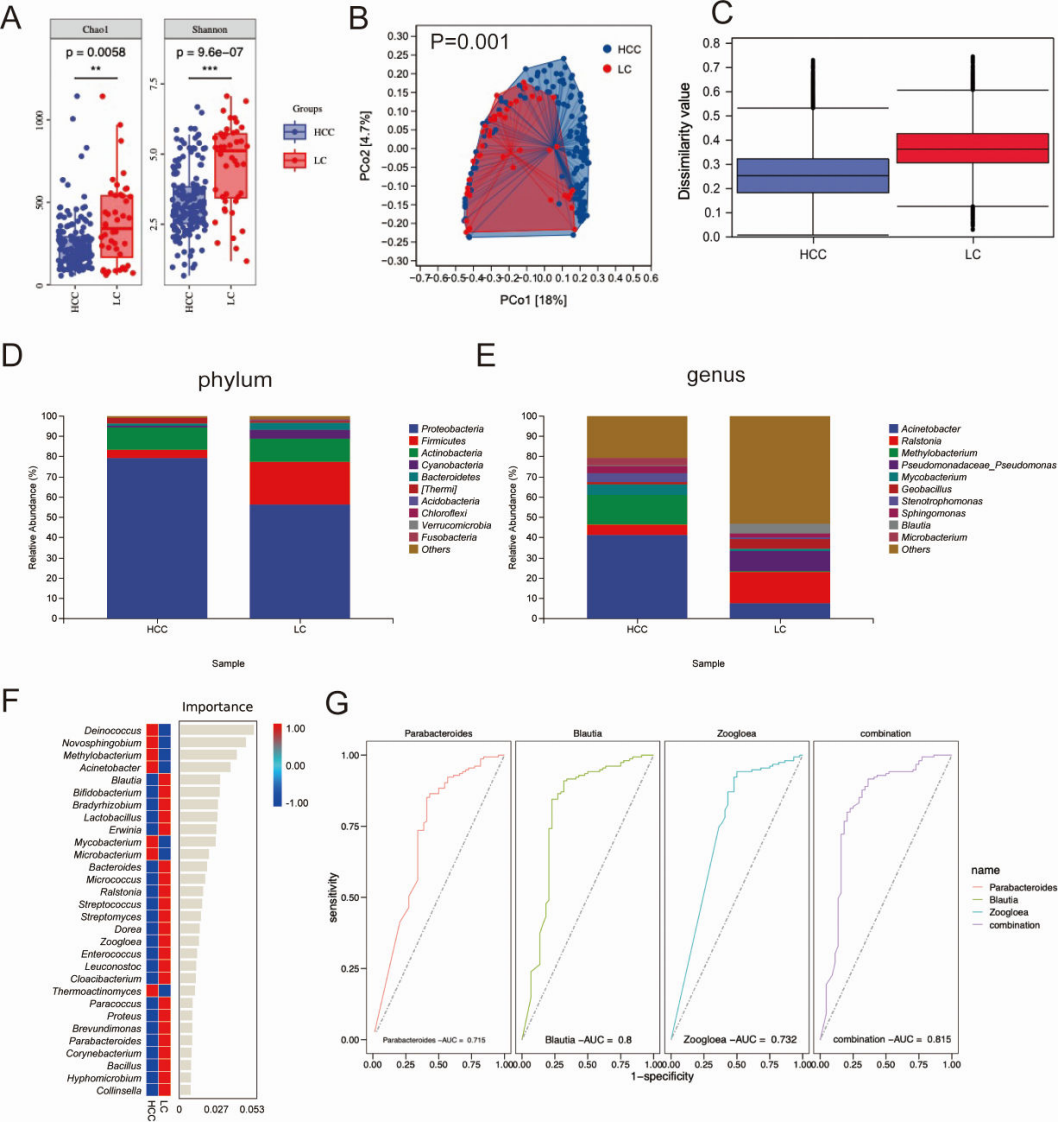
Comparison of the microbiomes between HCC and LC groups. (**A**) The microbial community richness and diversity (Chao1 richness estimator and Shannon diversity index). (**B**) PCoA analysis based on weighted UniFrac distance and (**C**) statistical analysis. The composition of ascites microbiota at the (**D**) phylum and (**E**) genus level. (**F**) Classification performance of the 30 most discriminant genera between the HCC and LC group by a random forest model and heatmap based on the relative abundance of the genera. (**G**) ROC curves and their corresponding AUCs of microbiome biomarkers for diagnosis of HCC. HCC: *n* = 196, LC: *n* = 44 for the research cohort. Two-sided Wilcoxon rank-sum test. *P < 0.05, **P < 0.01, and ***P < 0.001.

As α-diversity analysis is the general reflection of species richness, we applied this analysis to detect the microbiome richness difference between the HCC and LC groups. As shown in Fig. 2A, the Chao1 richness estimator (*P* = 0.0058) and Shannon index (*P* = 9.6e−07) demonstrated significant differences between the HCC and LC group, indicating that species richness was remarkably higher in the HCC than in the LC group. The β-diversity, calculated via principal coordinate analysis (PCoA), showed a significant difference between the HCC and LC groups: PCo1 is 18%, and PCo2 is 4.7% (Fig. 2B). The PCoA analysis is effective (*P* = 0.001; Fig. 2C). *Proteobacteria*, *Fusobacteria*, and *[Thermi]* were upregulated in the HCC group (Fig. 2D).*Acinetobacter*, *Methylobacterium*, *Mycobacterium*, *Sphingomonas*, *Stenotrophomonas*, and *Microbacterium* were upregulated in the HCC group (Fig. 2E). A random forest model was established, as shown in Fig. 2F, and a bar plot of the 30 genera with the highest ability to discriminate HCC from LC was computed at the genus level. Furthermore, receiver operating characteristic (ROC) analyses were performed to explore the diagnostic ability of significantly different bacteria. As shown in Fig. 2G, *Parabacteroides* [area under the curve (AUC) = 0.715], *Blautia* (AUC = 0.80), and *Zoogloea* (AUC = 0.732) exhibited good discriminative abilities, and the AUC of the combination of these three bacteria was 0.815.

Linear discriminant analysis effect size (LEfSe) is a comparative method for identifying biomarkers that can help differentiate HCC from LC. As listed in Fig. S2A and B, LEfSe showed a remarkable difference in species diversity between the HCC and LC groups, as detected using the Wilcoxon rank-sum test, and the cutoff value of linear discriminant analysis (LDA) was set at 3.28. A total of 100 species were identified; 24 were enriched in the HCC group, and 76 were enriched in the LC group.

To analyze the functional pathways related to significant microbiota, the 16S rRNA sequencing results were analyzed using the phylogenetic investigation of communities by reconstruction of unobserved states (PICRUSt2) module, similar to the results from the MetaCyc database. As shown in Fig. S3A and B, the top five biosynthesis genes were amino acid biosynthesis; cofactor, prosthetic group, electron carrier, and vitamin biosynthesis; nucleoside and nucleotide biosynthesis; fatty acid and lipid biosynthesis; and carbohydrate biosynthesis. Moreover, Table S2 lists the 20 most significant biological pathways identified using the MetaCyc database.

### Influence of clinical characteristics on HCC prognosis

**Fig 3:**
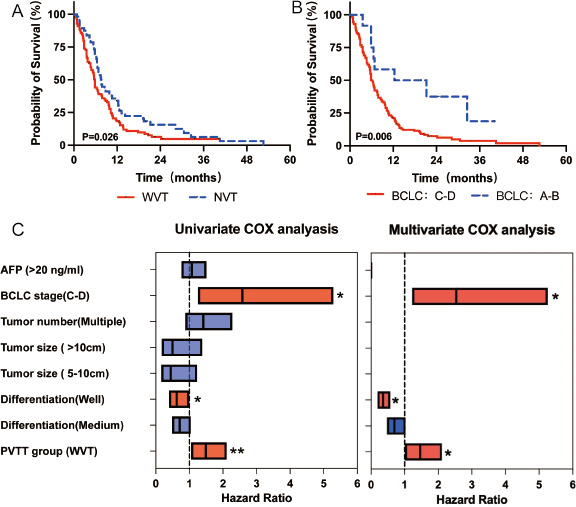
Survival analysis of patients with HCC. Survival curves of overall survival in subgroups: (**A**) WVT vs NVT group and (**B**) BCLC stage: A–B vs C–D. (**C**) HRs based on univariate and multivariate Cox analysis are exhibited. For this part of the research cohort, WVT: *n* = 125 and NVT: *n* = 71. The *P*-value of HR was calculated by Cox analysis. *P < 0.05, **P < 0.01, and ***P < 0.001.

To explore prognostic risk factors, Kaplan–Meier and Cox analyses were conducted in the HCC cohort. As shown in Fig. 3A and B, Kaplan–Meier analysis revealed that the WVT group (with PVTT, *n* = 125) had a poorer prognosis than the NVT group (non-PVTT, *n* = 71), and the BCLC stage C–D group had a poorer prognosis than the stage A–B group. Figure 3C shows the predictive power of PVTT for overall survival (Hazard ratio (HR) = 1.460, 95% CI: 1.011–2.108, *P* = 0.044). The detailed results of the Cox regression analysis are displayed in Table S3.

### Relationship between PVTT and ascitic microbiome

The clinical characteristics of 196 patients with HCC with subgroups are shown in Table S4. After the χ^2^ test, we found that PVTT correlated with the Child-Pugh score of the patients (*P* = 0.010).

**Fig 4:**
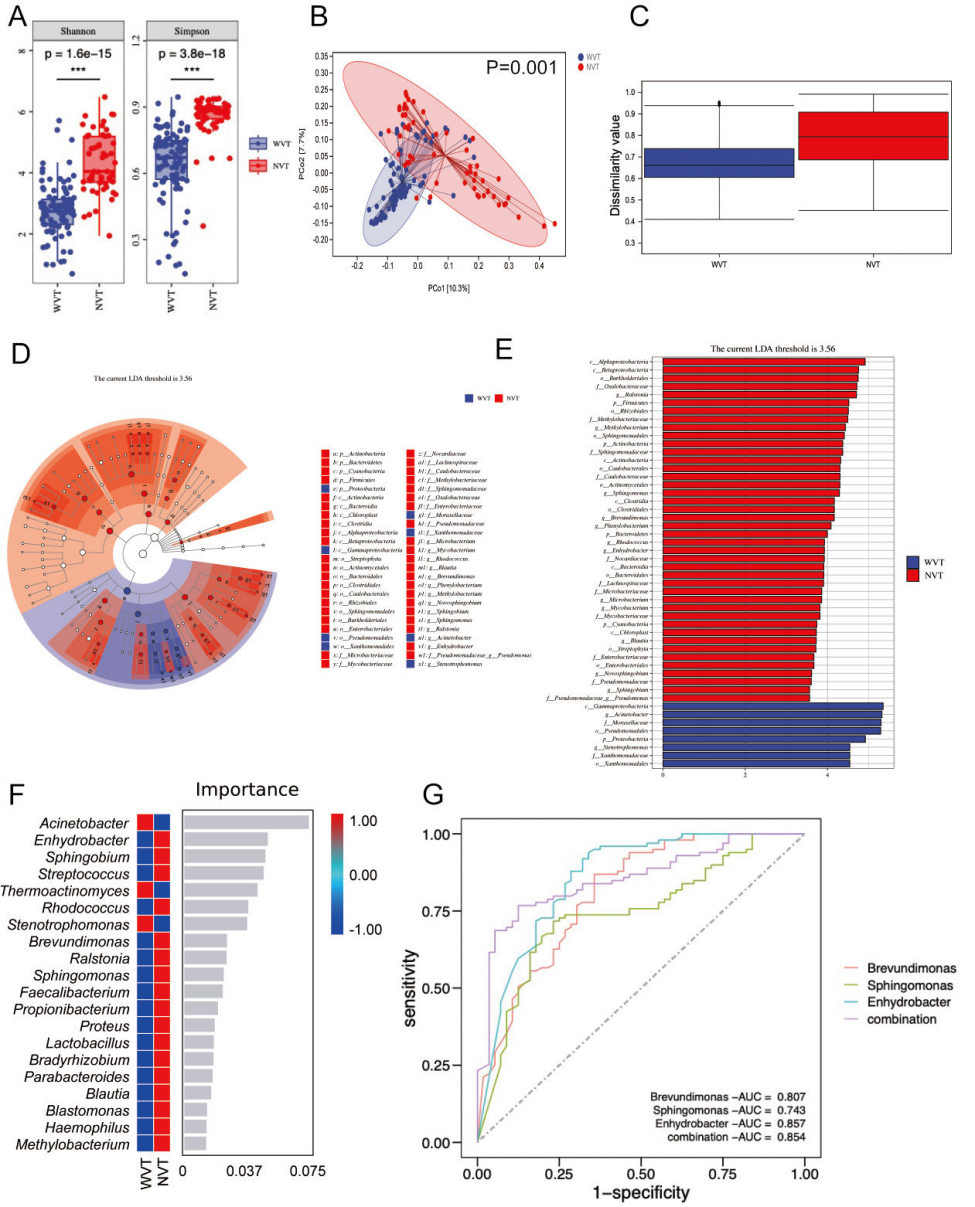
Characterization of the microbiome in WVT and NVT group. (**A**) Boxplot of Shannon diversity index and Simpson index. (**B**) PCoA based on unweighted UniFrac distance and (**C**) inner-group distance by analysis of similarities (*P* = 0.001). LEfSe analysis shows a huge difference in species diversity between the two groups. (**D**) Taxonomic cladogram of the LEfSe analysis. Each node represents a specific taxon (p, phylum; c, class; o, order; f, family; g, genus). White nodes denote the taxonomic features that are not significantly differentiated between WVT and NVT samples. Blue nodes denote the taxonomic types with more abundance in the WVT group than in NVT tissues, while red nodes represent the taxonomic types more abundant in the NVT group. (**E**) Histogram of LDA score of taxa with differential abundance between tumor and normal tissues. Only features with LDA score (log10) >3.56 and *P* < 0.05 are shown. (**F**) Classification performance of the 20 most discriminant genera between the two groups by a random forest model and heatmap based on the relative abundance of the genera. (**G**) ROC curves and their corresponding AUCs of microbiome biomarkers for predictive of WVT.

Subsequently, 155 patients with HCC with FFPE samples were divided into two groups: WVT (*n* = 99) and NVT (*n* = 56). As shown in Fig. 4A, the Shannon diversity index (*P* = 1.6e−15) and the Simpson index (*P* = 3.8e−18) were significantly higher in the NVT than in the WVT group. PCoA based on unweighted UniFrac distance metrics displayed a significant difference in the microbial community structure between the WVT and NVT groups: PCo1 was 9.1%, and PCo2 was 4.7% (Fig. 4B). Statistical analysis revealed that the PCoA analysis was effective (*P* = 0.001, using analysis of similarities; Fig. 4C). At the phylum level (Fig. S4A), the WVT group was characterized by significantly higher *Proteobacteria* and *Acidobacteria* than those in the NVT group. At the genus level (Fig. S4B), WVT was characterized by an expansion in many genera within *Acinetobacter*, *Stenotrophomonas*, *Bacillus*, and by a decrease in genera such as *Sphingomonas*, *Methylobacterium, and Ralstonia.*

As shown in Fig. 4D and E, LEfSe analysis demonstrated a significant difference in species diversity between the WVT and NVT groups, as detected using the Wilcoxon rank-sum test. A total of 50 species at the genus level were identified in the two groups when the cutoff value of LDA was set at 3.56. Eight gut bacteria were enriched in the WVT group, including *Proteobacteria*, *Pseudomonadales*, and *Acinetobacter*, and 42 species at the genus level were enriched in the NVT group. Using a random forest analysis, 20 genera were selected as the key genera that provided the best discriminatory power (Fig. 4F). The top three key genera showed reliable distinguishing ability in WVT, with AUC values of 0.807 (*Brevundimonas*), 0.743 (*Sphingomonas*), and 0.857 (*Enhydrobacter*; Fig. 4G); the AUC of the combination of these three bacteria was 0.854.

We identified the metabolic pathways that were significantly different between the groups using the metagenomeSeq R package. As shown in Fig. S5, the relative abundances of functional pathways were identified from the Kyoto Encyclopedia of Genes and Genomes (KEGG) database. The top five pathways of metabolism were amino acid metabolism, carbohydrate metabolism, metabolism of other amino acids, xenobiotics biodegradation and metabolism, and lipid metabolism. KEGG Orthology analysis revealed the top 20 significant metabolic pathways between the WVT and NVT groups (Table S5).

### Correlation between microbial signatures and clinical characteristics

**Fig 5:**
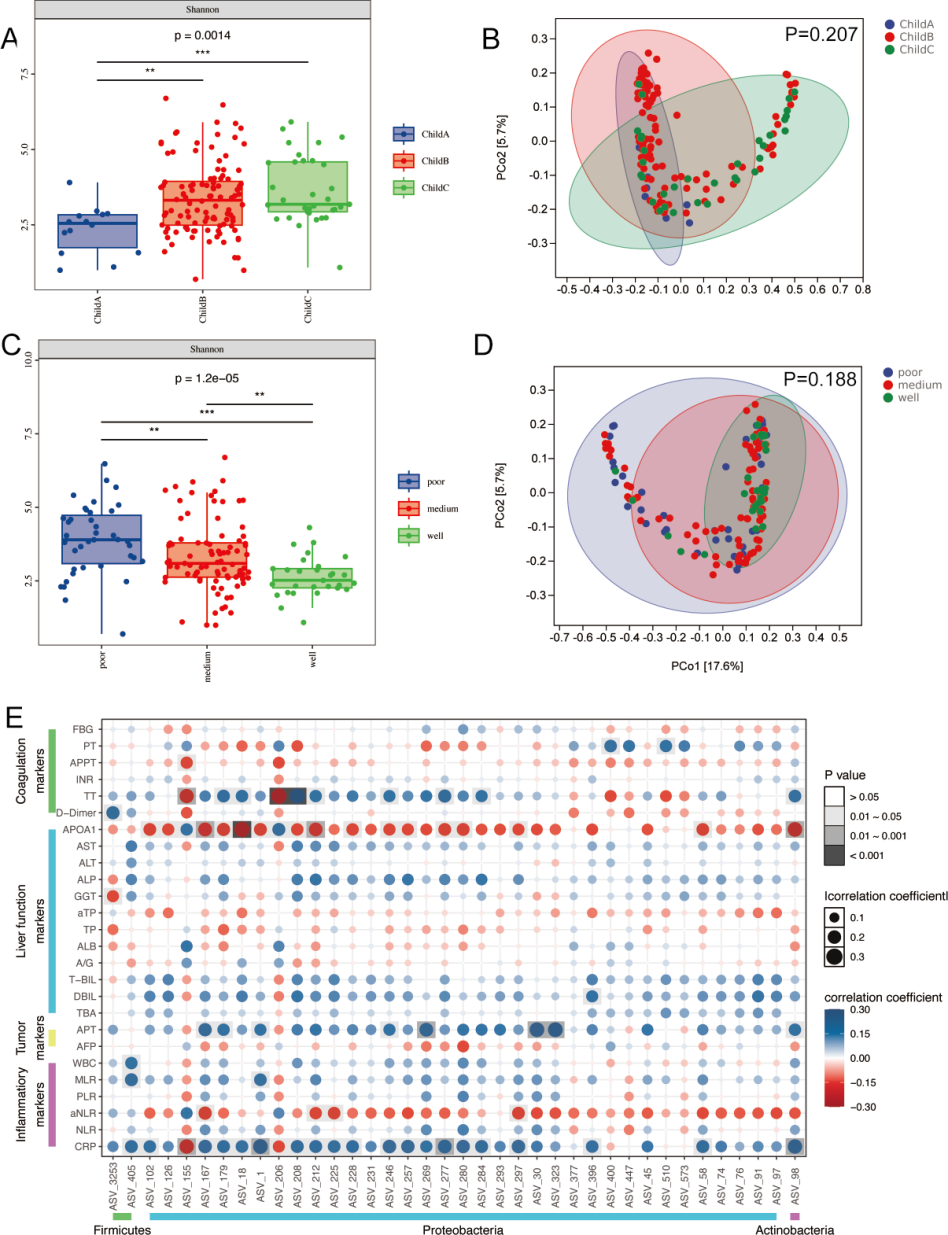
Correlations between the ascites microbiome with clinical characteristics. (**A–D**) The α-diversity and β-diversity of FFPE of malignant ascites are categorized by BCLC stage and tumor differentiation. (**E**) Heatmap indicating the correlations between the differential microbiomes of the WVT group (related to Fig. 4F) and clinical variables, including coagulation markers, liver function markers, tumor markers, and systemic inflammatory markers, derived from routine blood tests. FBG: fibrinogen; PT: prothrombin time; APTT: activated partial thromboplastin time; INR: international normalized ratio; TT: thrombin time; APOA1: apolipoprotein A1; AST: aspartate aminotransferase; ALT: aspartate aminotransferase; ALP: alkaline phosphatase; GGT: alkaline phosphatase; aTP: ascites total protein; TP: total protein; ALB: albumin; A/G: albumin-globulin ratio; T-Bil: total bilirubin; DBil: direct bilirubin; TBA: total bile acid; APT: Abnormal prothrombin; AFP: alpha-fetoprotein; WBC: white blood cell; MLR: monocyte-to-lymphocyte ratio; PLR: platelet-to-lymphocyte ratio; aNLR: ascites neutrophil-to-lymphocyte ratio; CRP: C-reactive protein. For this part of the research cohort, WVT: *n* = 99, NVT: *n* = 56. Two-sided Wilcoxon rank-sum test. *P < 0.05, **P < 0.01, and ***P < 0.001.

The relationship between microbial and clinical features (such as tumor differentiation, Child-Pugh class, and serum markers) was analyzed. As shown in Fig. 5A and C, there are significant differences in α-diversity among different Child-Pugh classes and tumor differentiation. The results revealed no significant difference in β-diversities among these classification methods (Fig. 5B and D).

Differential analysis was conducted between the WVT and NVT groups to identify the ASVs significantly dysregulated. Pearson’s analysis was performed to investigate the relationship between differential ASVs and biochemical features (Fig. 5E). Firmicutes were associated with elevated C-reactive protein, monocyte-to-lymphocyte ratio, white blood cell, and D-dimer levels. Proteobacteria were associated with the elevation of abnormal prothrombin and a reduction in the ascites neutrophil-to-lymphocyte ratio and apolipoprotein A1, indicating altered lipid metabolism.

### Comparison of immune cells in the WVT and NVT groups

**Fig 6:**
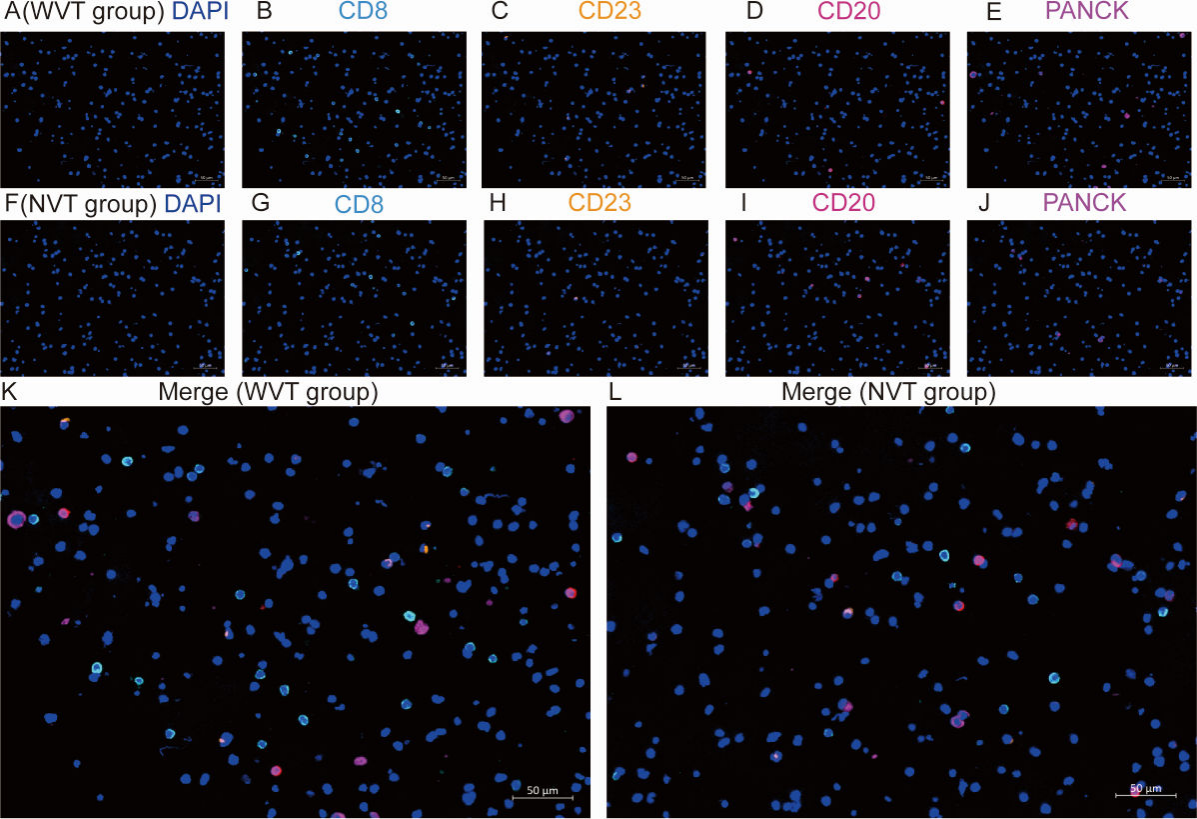
Five-color fluorescent mIHC analysis of tumor microenvironment from malignant ascites in the HCC individuals. The WVT group: (**A**) DAPI, (**B**) CD8, (**C**) CD23, (**D**) CD20, and (**E**) PANCK, and the NVT group: (**F**) DAPI, (**G**) CD8, (**H**) CD23, (**I**) CD20, and (**J**) PANCK, the merged picture of five markers after multispectral imaging of the (**K**) WVT group and (**L**) NVT group.

There are few reports on the relationship between PVTT and the tumor microenvironment, and fluorescent multiplex immunohistochemistry (mIHC) was used to detect the expression of different immune cells by labeling them with five markers, including DAPI. An ascite tissue microarray was constructed using 24 FFPE samples (WVT, *n* = 15; NVT, *n* = 9). Representative fluorescence images from the WVT and NVT groups are shown separately in Fig. 6. Four common immune cells were qualified and compared between the WVT and NVT groups (Fig. S6). Statistical analyses revealed no significant differences in the densities of CD8+T, CD23, CD20, and PANCK cells between the WVT and NVT groups.

## DISCUSSION

Malignant ascites remains a bottleneck for treatment. The potential role of the ascites microbiota in disease progression has not been well explored. This is the first study to investigate the differences in the ascites microbiota between ascites secondary to HCC and to LC based on 16S rRNA. PVTT is a prognostic marker in patients with HCC with ascites. The results of 16S rRNA sequencing based on 155 FFPE samples showed significantly decreased microbial richness and evenness in patients with HCC with PVTT compared with those without PVTT. The relative abundances of *Proteobacteria* and *Acidobacteria* were significantly elevated in patients with HCC with PVTT. We explored the relationship between PVTT and tumors using an mIHC assay. Some high-impact studies have collected FFPE as experimental samples to explore the relationship between tumor microbiota and cancer development, providing further support for the reliability of our approach and results (10). Cao et al. (11) collected 166 paraffin-embedded tumor tissues and 24 frozen tumor tissues to investigate the prognostic effects of the systemic inflammation response index on the survival of colorectal cancer patients. Similar results were obtained in 16S rRNA based on the two different samples. Taken together, our study provides novel insights into the association between ascitic microbiome and PVTT in HCC, which is important for clinical treatment.

Differentiating malignant ascites from benign ascites has always been a clinical challenge, and looking for specific markers is one of the “hot spots” in the research. Previous studies have focused on serum or ascites biomarkers, and no studies have examined markers from the perspective of microbiota. Wang et al. (12) constructed a plasmonic nanocavity-based electrochemiluminescence sensor to detect exosomal miRNA-223–3p in ascites and assisted in the early diagnosis of peritoneal metastasis of gastric cancer. Wang et al. (13) developed an extracellular vesicle miRNA signature in ovarian cancer with high diagnostic accuracy to differentiate benign peritoneal fluids from malignant ascites (in the ascites training and validation sets, AUC = 1.0). Approximately 40% of people with cirrhosis are diagnosed when they present with complications such as ascites or esophageal varices (14). Malignant ascites frequently develops in patients with advanced HCC and is associated with decreased long-term survival (3). Hanafy et al. revealed that ascites calprotectin may be an easy marker for predicting the early occurrence of HCC (15). We identified the differentially expressed microbiota between the ascites secondary to the HCC group and those secondary to the LC group. Hence, three differentiated microbiota were selected and showed good discriminative ability, including *Parabacteroides* (AUC = 0.715), *Blautia* (AUC = 0.80), and *Zoogloea* (AUC = 0.732). The AUC of the combination of these three bacteria was 0.815. Further studies are required to fully investigate the influence of these microbiota on HCC progression.

Tumor microbiota plays a vital role in the progression and survival outcomes of HCC (7). A “gut-liver axis” facilitates bidirectional communication between intestinal microbes and bile acids, playing a pivotal function in HCC pathogenesis(16,17). The healthy epithelium forms a tightly sealed physical barrier that separates the host from the intestinal microbiome, and dysbiosis and microbiome disorders can affect gut barrier function (18). The permeability of the intestinal mucosa increases, allowing the translocation of viable bacteria to cross and enter the blood or abdominal cavity (19). Ascites reflects the tumor microenvironment and act as a pro-inflammatory reservoir for numerous soluble proteins, making ascites the perfect microenvironment to promote tumor cell metastasis (20, 21). We speculated that changes in the intestinal microbiota can affect the microbiota community of malignant ascites, affecting the immune status in the malignant ascites microenvironment and thereby affecting the survival of patients with HCC. The gut microbiota closely shapes the hepatic inflammatory microenvironment and opens up new cancer prevention and therapy approaches (8). High dietary fructose promotes HCC progression through microbial acetate-induced hyperO-GlcNAcylation (22). However, the potential function of microbiota in malignant ascites (secondary to HCC) is not fully understood. Peritoneal metastasis is the leading cause of death in HCC, and malignant ascites is caused by the intraperitoneal spread of solid tumor cells (23). Kansuiphorin C and kansuinin A can ameliorate malignant ascites by modulating the gut microbiota, including an increase in *Lactobacillus* and a decrease in *Helicobacter* and related carbohydrate and amino acid metabolism (24). Therefore, it is imperative to identify a new entry point from the ascitic microbiota to burdensome ascites-associated symptoms and hospitalization rates. This study found a significant difference in microbiota evenness and diversity between the HCC and LC groups. PICRUSt2 module demonstrated that fatty acid and lipid biosynthesis may be key metabolic pathways that deserve further exploration.

Many factors are associated with the prognosis of HCC, and the prognosis of patients with HCC and PVTT is much worse than that of patients without PVTT (5). PVTT is a common complication in patients with advanced HCC and has captured the attention of clinicians (24). Our findings are consistent with those of previous reports, and the results of the Cox analysis indicated that BCLC stage and PVTT are independent risk factors for HCC prognosis. The differential abundance of microbiota in the WVT and NVT groups was associated with coagulation markers (abnormal prothrombin, D-dimer, and thrombin time). PVTT progression has many characteristics, including complications related to portal hypertension, worse liver function, and poor tolerance to treatment, which may be associated with poor prognosis (25). Fecal microbiota transplantation is a safe method for improving the short- and medium-term survival of patients with liver disease (26). Hence, a better understanding of the relationship between PVTT and the gut microbiota in HCC is important for its clinical management. *Proteobacteria* are widely detected in the gut microbiota of patients with HCC. Similar to these findings, we found that the abundance of *Proteobacteria* was significantly higher in the WVT group than in the NVT group. Previous studies have demonstrated that increased *Proteobacteria* abundance is likely related to the risk of cachexia and poor prognosis (27, 28). *Acinetobacter* is an important opportunistic pathogen (29), and *Stenotrophomonas* is frequently found in patients with cancer (30). We found that the abundance of *Acinetobacter* and *Stenotrophomonas* was higher in the WVT group. Our study highlights the importance of exploring PVTT-associated microbial features at the functional and metabolic levels to identify new potential therapeutic targets.

The gut-liver-immune axis describes a unique relationship between the intestinal microbiome, liver, and mucosal immune system (31). In addition to being blood-based, platelet-tumor cell interactions may occur in ascites and the tumor microenvironment (32). Irina et al. (33) reported that ovarian cancer tissues and ascites contain lymphocytic infiltrates inhibited by immunosuppressive molecules within the tumor microenvironment. Previous studies have shown that the potential molecular mechanisms underlying PVTT involve an immunosuppressive microenvironment (34), deregulatory noncoding RNA (35), epigenetic regulation (36), hypoxia (37), and viral hepatitis (38). Yang et al. reported that tumor growth factor-beta can increase the chemokine C-C motif chemokine 22 level, which attracts regulatory T cells to facilitate immune escape and increase colonization of disseminated HCC cells in the portal vein (39). Our findings differ from previous findings in that there were no significant differences in the densities of the four selected immune cells between the WVT and NVT groups. This may be partly due to the small mIHC sample size and limited immune markers (CD13, CD24, and CD44), and some reported markers were not selected in our study. Spearman’s correlation analysis revealed that the differences in ASVs were mainly correlated with the clinical features of apolipoprotein A1 and C-reactive protein. Our analysis utilized the PICRUSt2 module to investigate different potential functional pathways and showed that lipid metabolism may be involved in the development of PVTT, which is consistent with the results of the correlation analysis. Hence, our study sheds light on the hypothesis that these key ascites microbiota affect PVTT progression by regulating lipid metabolism in individuals.

Compared with recent studies related to malignant ascites, the strength of this study is that it displayed some novel insights and has potential translational value. However, this study had several limitations. First, the sample size and types were limited, particularly in mIHC groups. Due to the limited sample size and sample types, the results should be interpreted with caution. Second, this was a single-center study, and the predictive value of microbiota markers in patients with ascites secondary to HCC must be verified using an external validation cohort. Third, the technical means of metagenomics and shotgun sequencing could offer more comprehensive experimental data compared with 16S rRNA, but FFPE samples are more suitable for 16S rRNA sequencing because of the retrospective nature of this study. Finally, the number of immune markers in the mIHC that we selected was limited, and the hidden link between PVTT and immune status may not have been demonstrated. Hence, more robust basic and translational studies are needed on the mechanisms by which the microbiome influences the tumor microenvironment in malignant ascites.

In conclusion, this is the first clinical study based on FFPE samples to investigate ascitic microbiome differences between malignant ascites secondary to HCC and benign ascites secondary to LC. Microbiome analysis revealed a significant difference in ascitic microbiome between HCC and LC groups. We then characterized the microbiomes in the WVT and NVT groups and discovered differences in microbial diversity between these two groups. Thus, microbiome analysis can aid in predicting PVTT formation and may provide a crucial basis for a better understanding of PVTT therapy in patients with HCC.

## MATERIALS AND METHODS

### Subjects and sample collection

A total of 1,686 patients with ascites who underwent diagnostic abdominal paracentesis at Wuhan University of Renmin Hospital from January 2017 to May 2023 were initially sifted, and 240 subjects were included in this study. A total of 199 FFPE tissues of ascites samples (malignant ascites secondary to HCC = 155 and benign ascites secondary to LC = 44) and 41 patients’ clinical information was collected without sample collection.

The inclusion criteria for patients with ascites secondary to HCC were as follows (40, 41): (i) patients were pathologically diagnosed with HCC and were confirmed by examination by one or more pathologists; (ii) patients were diagnosed with peritoneal fluid and underwent abdominal paracentesis; (iii) tumor cells were found in the peritoneal fluid by cytological examination; (iv) patients’ clinical data are available in the Electronic Medical System. The exclusion criteria for patients with ascites secondary to HCC were as follows: (i) patients with benign ascites, such as renal insufficiency, and hypoproteinemia; (ii) patients with pleural effusion; (iii) patients with a history of other cancers within the past 5 years; (iv) patients with false-malignant ascites after a combined course of chemotherapy; (v) patients with incomplete clinical information.

The diagnostic criteria for LC have been described previously (42); the rest are based on liver histology, which shows progressive liver fibrosis. The entry criteria for the LC group included patients with benign ascites secondary to LC without critical clinical information missing.

Demographic data, laboratory test results, and therapeutic information were collected from the Electronic Medical System in a standard format. Patients were followed up through the death date or the last follow-up date, 31 May 2023; mortality follow-up was more than 99% complete. Survival information was obtained from several methods—medical records, semiannual clinical visits, and/or quarterly telephone interviews with patients or patient families, including the development of metastases and treatments. Overall survival was defined as the time to death from the start of treatment. Survival status (dead or alive) and overall survival time (months) were recorded by the researcher.

### Diagnosis of HCC with PVTT

Individuals with HCC who fulfilled the entry criteria for the WVT (with PVTT) group were sorted into the WVT group, and the remaining individuals with HCC were sorted into the NVT (non-PVTT) group. The standard diagnosis of HCC with PVTT relied on the typical imaging findings of a hepatic mass (i.e., early arterial enhancement with early port venous washout) and a coexisting portal vein thrombus with continuity (43). The inclusion criteria for the WVT group were as follows: patients were diagnosed with PVTT according to the diagnostic criteria by more than two imaging specialists via detailed imaging.

### DNA extraction and 16S rDNA gene amplicon sequencing

The Omega Mag-Bind soil DNA Kit (Omega Bio-Tek, Norcross, GA, USA) was used to extract total microbial DNA from the selected samples. The quantity and quality of extracted DNA were assessed using a NanoDrop NC2000 spectrophotometer (Thermo Fisher Scientific, Waltham, MA, USA) and agarose gel electrophoresis, respectively. The bacterial 16S rRNA genes V3–V4 regions were amplified using the forward primer 338F (5′-ACTCCTACGGGAGGCAGCA-3′) and the reverse primer 806R (5′-GGACTACHVGGGTWTCTAAT-3′). The PCR amplicons were purified using Vazyme VAHTSTM DNA Clean Beads (Vazyme, Nanjing, China). After purification, the final amplicon was quantified using a Quant-iT PicoGreen dsDNA Assay Kit (Invitrogen, Carlsbad, CA, USA). After the individual quantification steps, the purified amplicons were pooled in equal amounts, and 16S rRNA gene sequencing of the sample DNA was performed using the Illumina MiSeq platform with the MiSeq Reagent Kit v3 at Bioyi Biotechnology Co., Ltd. Wuhan, China.

### Microbiome analysis

Microbiome bioinformatics of the raw sequences was processed using QIIME2 2019.4, with slight modifications, according to the official tutorials (http://docs.qiime2.org/2019.4/tutorials/). Non-singleton ASVs were aligned using mafft (44) and used to construct a phylogenetic tree using fasttree2. A rarefaction curve was used to estimate the richness of the ASV. The Simpson index, Chao1 richness estimator, Shannon diversity index, Goods coverage, Pielou’s evenness, Faith’s PD, and Observed species were used to assess alpha diversity between different groups (HCC vs LC and WVT vs NVT) at the ASV level. PCoA was performed to evaluate the beta diversity among the gut microbial communities in different individuals. A Venn diagram was performed to visualize the shared and unique ASV among samples or groups using the R package “VennDiagram,” based on the occurrence of ASVs. The LEfSe method was employed to filter out significantly abundant taxa of bacteria (LDA score >3) between the different groups at the phylum and genus levels. Orthogonal Partial Least Squares Discriminant Analysis was utilized as a supervised model to reveal the microbiota variation among groups, using the R package “muma.” Random Forest analysis was applied to discriminate the samples from different groups using QIIME2 with default settings. The functions of significant microbiota between different groups were predicted using CoG and PICRUSt2 based on KEGG and MetaCyc databases (http://metacyc.org/).

### Fluorescent mIHC

To determine the association between PVTT and the tumor microenvironment, fluorescent mIHC was performed using an AlphaTSA Multiplex IHC Kit (Alpha X Biotech Co., LTD, Beijing, China). The FFPE slides were deparaffinized in xylene, rehydrated in graded alcohol, retrieved in EDTA buffer, and blocked using antibody diluent. Subsequently, the FFPE slides were incubated with primary antibodies for 1 h and second antibodies for 10 min at 37°C. Fluorescent dyes from the AlphaTSA Multiplex IHC Kit were used for fluorescence staining. Heat-induced epitope retrieval was performed to clear primary antibodies after each staining cycle. Finally, the FFPE slides were stained with DAPI for five minutes, followed by enclosure in Antifade Mounting Medium. The FFPE slide was fully scanned and imaged for subsequent analysis using ZEN software (version 3.3). The primary and secondary antibodies and their relevant information are listed in Table S1.

### Statistical analysis

The data were processed using Microsoft Excel 2019, SPSS software (version 27.0, SPSS Inc., Chicago, IL, USA), and GraphPad Prism (version 9.5.1). R software (version 3.0) was used to draw the figures. Differences between the WVT and NVT groups were calculated using the χ^2^ test or Fisher’s exact test. Survival was analyzed using Kaplan–Meier curves, the log-rank test, and Cox regression. ROC curves were utilized to filter out microbiome biomarkers for different groups (HCC vs LC and WVT vs NVT), and the AUC was commonly used to assess discriminative power. An AUC value close to 1 (AUC > 0.8) indicated good classifier variables. For correlation analysis between microbial signatures and clinical characteristics, Spearman’s correlation was calculated and performed on the R software package of “ggplot2,” “RColorBrewer,” and “reshape2.” R coefficient was calculated to measure the relationship between microbial signatures and clinical characteristics, and R coefficient was divided into three types (week: 0.2–0.4, medium: 0.4–0.6, and strong: >0.6). Statistical parameters, including the exact value of *n* and statistical significance (*P*-value), are shown in the Figure Legends. Statistical significance was set at a two-sided *P*-value < 0.05.

## Data Availability

The datasets used during the current study are available from the corresponding author upon reasonable request.

## SUPPLEMENTAL MATERIAL

Supplemental material mbio.00245-24-s0001.docx Tables S1–S5; Fig. S1–S6.

ASM does not own the copyrights to Supplemental Material that may be linked to, or accessed through, an article. The authors have granted ASM a non-exclusive, world-wide license to publish the Supplemental Material files. Please contact the corresponding author directly for reuse.
