# Supplementary material for: Ascitic microbiota alteration is associated with portal vein tumor thrombosis occurrence and prognosis in hepatocellular carcinoma
Source: mBio. 2024 Apr 2;15(5):e00245-24. doi: 10.1128/mbio.00245-24 (PMC11077998; doi:10.1128/mbio.00245-24)
Supplement: Supplemental material — Tables S1–S5; Fig. S1–S6. [file mbio.00245-24-s0001.docx]

**Supplemental information**

**Intratumoural microbiome analyses define bacteria-﻿Portal vein tumor thrombosis interplay and microbial markers of hepatocellular carcinoma**

Yingyun Guo, ShanTian, Na zhan, Chuan Liu , Jiao Li, Jiaming Hu, Meiqi Qiu, Shuo Wang, Weiguo Dong

**The file includes:**

**SupplementaryTable 1.** The detailed information of the primary and second antibodies in this research

**SupplementaryTable 2**. MetaCyc metabolic pathways (top 20) in HCC and LC groups.

**SupplementaryTable3**. COX regression analysis for Overall Survival prediction.

**SupplementaryTable****4**. ﻿Comparisons of clinical characteristics between ascites secondary to WVT and NVT groups.

**SupplementaryTable5.** KEGG Orthology metabolic pathways (top 20) in WVT and NVT groups.

**SupplementaryFigure1.** Rarefaction curve and gut microbiota composition between HCC and LC group. **﻿(A)** Rarefaction curve. **(B)** The Venn plots reveal the unique and common taxa between HCC and LC groups.

**SupplementaryFigure2.** Linear discriminant analysis effect size (LEfSe) analysis, depicting the differential taxa between the microbiome of HCC and LC. **(A)** Taxonomic cladogram of the LEfSe analysis. Each node represents a specific taxon (p, phylum; c, class; o, order; f, family; g, genus). White nodes denote the taxonomic features that are not significantly differentiated between HCC and LC. Blue nodes denote the taxonomic types with more abundance in HCC, while red nodes represent the taxonomic types more abundant in LC. **(B)** Histogram of linear discriminant analysis (LDA) score of taxa with differential abundance between HCC and LC. Only features with LDA score (log 10) > 2.0 and P < 0.05 are shown.

**SupplementaryFigure3.** (**A)**. Representative pathways from MetaCyc database between the HCC and LC groups. (**B)**. Significant biological pathways between the two groups based on MetaCyc database.

**SupplementaryFigure4.** The relative abundance of microbial taxa at **﻿(A)** phylum and **(B)** genus levels; phyla or genera with a relative abundance <1% in each sample are merged into others.

**SupplementaryFigure** **5.** (**A)**. Representative pathways from KEEG database between the WVT and NVT groups. (**B)**. Significant biological pathways between the two groups based on KEEG database.

**SupplementaryFigure 6.** Quantitative analysis of four immune cells in the WVT and NVT group. The density of **(A)** CD8 + T cells, **(B)** CD23+ cells, **(C)** CD20+ cells, **(D)** PANCK+ cells in the tissue of WVT and NVT groups.

**Supplementary Table 1**. The detailed information of the primary and second antibodies in this research

| **Antibody** | **PanCK** | **CD23** | **CD8** | **CD20** |
| --- | --- | --- | --- | --- |
| No. | ZM0069 | ZA-0516 | ZA0508 | ab78237 |
| Species | Mouse | rabbit | rabbit | rabbit |
| Concentration | 1:300 | 1:200 | 1:100 | 1:200 |
| Incubation condition | 37℃ 1hr | 37℃ 1hr | 37℃ 1hr | 37℃ 1hr |
| Dye (1:100) | XTSA780 | XTSA 570 | XTSA 480 | XTSA 620 |

**Supplementary Table 2**. MetaCyc metabolic pathways (top 20) in HCC and LC groups.

| **Pathway** | **description** | **logFC** | **SE** | **P-values** | **Adj P-values** |
| --- | --- | --- | --- | --- | --- |
| PWY-1622 | formaldehyde assimilation I (serine pathway) | 2.37 | 0.237 | 0 | 0 |
| P241-PWY | coenzyme B biosynthesis | 2.291 | 0.3643 | 3.22E-10 | 1.20E-08 |
| HCAMHPDEG-PWY | 3-phenylpropanoate and 3-(3-hydroxyphenyl)propanoate degradation to 2-oxopent-4-enoate | 2.29 | 1.406 | 0.1035 | 0.5629 |
| PWY-6690 | cinnamate and 3-hydroxycinnamate degradation to 2-oxopent-4-enoate | 2.29 | 1.406 | 0.1035 | 0.5629 |
| CRNFORCAT-PWY | creatinine degradation I | 2.154 | 0.3112 | 4.40E-12 | 2.38E-10 |
| PWY-5741 | ethylmalonyl-CoA pathway | 2.121 | 1.367 | 0.1207 | 0.6332 |
| PWY-7007 | methyl ketone biosynthesis | 1.92 | 0.2425 | 2.44E-15 | 2.72E-13 |
| GALLATE-DEGRADATION-II-PWY | gallate degradation I | 1.872 | 0.3061 | 9.65E-10 | 3.07E-08 |
| PWY0-1277 | 3-phenylpropanoate and 3-(3-hydroxyphenyl)propanoate degradation | 1.839 | 1.175 | 0.1177 | 0.6252 |
| PWY-5419 | catechol degradation to 2-oxopent-4-enoate II | 1.81 | 0.2205 | 2.22E-16 | 3.30E-14 |
| PWY-5531 | chlorophyllide a biosynthesis II (anaerobic) | 1.801 | 1.226 | 0.1418 | 0.6659 |
| PWY-7159 | chlorophyllide a biosynthesis III (aerobic, light independent) | 1.801 | 1.226 | 0.1418 | 0.6659 |
| PWY-6383 | mono-trans, poly-cis decaprenyl phosphate biosynthesis | 1.765 | 1.377 | 0.1999 | 0.7951 |
| LIPASYN-PWY | phospholipases | 1.763 | 0.3227 | 4.64E-08 | 1.29E-06 |
| PWY-7013 | L-1,2-propanediol degradation | 1.758 | 0.3326 | 1.25E-07 | 3.09E-06 |
| PWY-6948 | sitosterol degradation to androstenedione | 1.743 | 0.3452 | 4.41E-07 | 9.83E-06 |
| PWY-6397 | mycolyl-arabinogalactan-peptidoglycan complex biosynthesis | 1.682 | 0.3433 | 9.66E-07 | 1.96E-05 |
| PWY-5420 | catechol degradation II (meta-cleavage pathway) | 1.648 | 0.2089 | 3.11E-15 | 2.77E-13 |
| PWY-6944 | androstenedione degradation | 1.61 | 0.2629 | 9.28E-10 | 3.07E-08 |
| PWY-3801 | sucrose degradation II (sucrose synthase) | 1.5 | 0.4851 | 0.001984 | 0.02011 |

**Supplementa**r**y Table 3.** COX regression analysis for Overall Survival prediction.

| **Variables** | **Univariate analysis** | |  | **Multivariate analysis** | |
| --- | --- | --- | --- | --- | --- |
|  | **HR (95% CI)** | **P** |  | **HR (95% CI)** | **P** |
| PVTT group |  |  |  |  |  |
| NVT | Reference |  |  |  |  |
| WVT | 1.488(1.051-2.108) | **0.025** |  | 1.460(1.011-2.108) | **0.044** |
| Differentiation |  |  |  |  |  |
| Poor | Reference |  |  |  |  |
| Medium | 0.713(0.489-1.040) | 0.713 |  | 0.696(0.473-1.023) | 0.065 |
| Well | 0.622(0.391-0.991) | **0.046** |  | 0.571(0.355-0.919) | **0.021** |
| Tumor size (cm) |  |  |  |  |  |
| <5 | Reference |  |  |  |  |
| 5-10 | 0.445(0.161-1.230) | 0.119 |  | - |  |
| >10 | 0.497(0.178-1.385) | 0.181 |  | - |  |
| Tumor number |  |  |  |  |  |
| Single | Reference |  |  |  |  |
| Multiple | 1.416(0.881-2.275) | 0.151 |  | - |  |
| BCLC stage |  |  |  |  |  |
| A-B | Reference |  |  |  |  |
| C-D | 2.576(1.255-5.289) | **0.010** |  | 2.535(1.222-5.257) | **0.012** |
| AFP (ng/ml) |  |  |  |  |  |
| ≤20 | Reference |  |  | - |  |
| >20 | 1.074(0.766-1.506) | 0.678 |  |  |  |

AFP:Alpha fetoprotein

**Supplementary** **Table 4**. ﻿Comparisons of clinical characteristics between ascites secondary to WVT and NVT groups.

| **Characteristics** | **WVT (N=125)** | **NVT (N=71)** | **P** |
| --- | --- | --- | --- |
| Age |  |  |  |
| ≤ 60 | 46(36.8%) | 33(46.5%) | 0.184 |
| > 60 | 79(63.2%) | 38(53.5%) |  |
| Gender (male) |  |  |  |
| Male | 89(71.2%) | 52(73.2%) | 0.760 |
| Female | 36(28.8%) | 19(26.8%) |  |
| Smoking statues |  |  |  |
| Never | 80(64.0%) | 51(71.8%) | 0.263 |
| Current/former | 45(36.0%) | 20(28.2%) |  |
| HBV infection (yes) | 85(68.0%) | 43(60.6%) | 0.293 |
| Tumor number (multiple) | 109(87.2%) | 60(84.5%) | 0.599 |
| Tumor size |  |  |  |
| ≤5 | 20(16.0%) | 18(25.4%) | 0.091 |
| 5-10 | 58(46.4%) | 36(50.7%) |  |
| >10 | 47(37.6%) | 17(23.9%) |  |
| Tumor location |  |  |  |
| Left lobe | 34(27.2%) | 24(33.8%) | 0.139 |
| Right lobe | 8(6.4%) | 9(12.7%) |  |
| both | 83(66.4%) | 38(53.5%) |  |
| Differential grade |  |  |  |
| Poor | 39(31.2%) | 23(32.4%) | 0.851 |
| Medium | 57(45.6%) | 34(47.9%) |  |
| Well | 29(23.2%) | 14(19.7%) |  |
| BCLC stage |  |  |  |
| A-B | 10(8.0%) | 7(9.9%) | 0.657 |
| C-D | 115(92.0%) | 64(90.1%) |  |
| Child-Pugh grading |  |  |  |
| A | 14(11.2%) | 9(12.7%) | 0.010 |
| B | 78(62.4%) | 56(78.9%) |  |
| C | 33(26.4%) | 6(8.6%) |  |

**Supplementary Table 5**. KEGG Orthology metabolic pathways (top 20) in WVT and NVT groups.

| **pathway** | **description** | **｜logFC｜** | **SE** | **Pvalues** | **adjPvalues** |
| --- | --- | --- | --- | --- | --- |
| ko04310 | Wnt signaling pathway | 4.739 | 1.015 | 3.041E-06 | 0.00009883 |
| ko04110 | Cell cycle | 4.084 | 1.158 | 0.0004221 | 0.006516 |
| ko00901 | Indole alkaloid biosynthesis | 3.155 | 1.048 | 0.002596 | 0.02531 |
| ko03022 | Basal transcription factors | 2.462 | 0.5941 | 0.00003421 | 0.0009323 |
| ko04080 | Neuroactive ligand-receptor interaction | 2.457 | 0.438 | 2.01E-08 | 1.308E-06 |
| ko04320 | Dorso-ventral axis formation | 2.403 | 1.322 | 0.06917 | 0.4262 |
| ko04075 | Plant hormone signal transduction | 2.355 | 0.6293 | 0.0001829 | 0.003567 |
| ko04962 | Vasopressin-regulated water reabsorption | 2.248 | 0.33 | 9.75E-12 | 9.50E-10 |
| ko00940 | Phenylpropanoid biosynthesis | 2.221 | 0.6385 | 0.0005045 | 0.006559 |
| ko01057 | Biosynthesis of type II polyketide products | 1.793 | 0.3245 | 3.28E-08 | 0.0000016 |
| ko04740 | Olfactory transduction | 1.744 | 1.658 | 0.2928 | 0.9669 |
| ko03015 | mRNA surveillance pathway | 1.621 | 0.4383 | 0.000217 | 0.003846 |
| ko00601 | Glycosphingolipid biosynthesis - lacto and neolacto series | 1.571 | 0.695 | 0.0238 | 0.2017 |
| ko00401 | Novobiocin biosynthesis | 1.557 | 0.7211 | 0.03081 | 0.2311 |
| ko00196 | Photosynthesis - antenna proteins | 1.556 | 0.4539 | 0.000609 | 0.007422 |
| ko00524 | Butirosin and neomycin biosynthesis | 1.413 | 0.3431 | 0.00003825 | 0.0009323 |
| ko05100 | Bacterial invasion of epithelial cells | 1.377 | 1.391 | 0.3222 | 0.9669 |
| ko04512 | ECM-receptor interaction | 1.362 | 0.6924 | 0.04922 | 0.331 |
| ko05010 | Alzheimer's disease | 1.332 | 0.431 | 0.001998 | 0.02164 |
| ko00472 | D-Arginine and D-ornithine metabolism | 1.266 | 0.9692 | 0.1916 | 0.8303 |


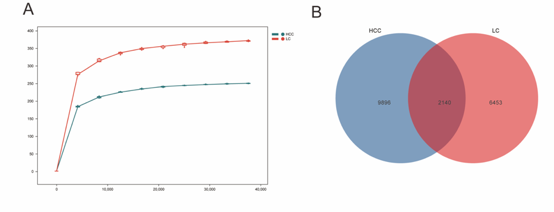


**Supplementary Figure1.** Rarefaction curve and gut microbiota composition between HCC and LC group. **﻿(A)** Rarefaction curve. **(B)** The Venn plots reveal the unique and common taxa between HCC and LC groups.


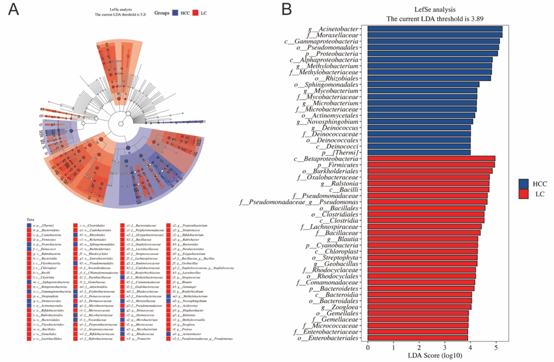


**Supplementary Figure 2**. Linear discriminant analysis effect size (LEfSe) analysis, depicting the differential taxa between the microbiome of HCC and LC. **(A)** Taxonomic cladogram of the LEfSe analysis. Each node represents a specific taxon (p, phylum; c, class; o, order; f, family; g, genus). White nodes denote the taxonomic features that are not significantly differentiated between HCC and LC. Blue nodes denote the taxonomic types with more abundance in HCC, while red nodes represent the taxonomic types more abundant in LC. **(B)** Histogram of linear discriminant analysis (LDA) score of taxa with differential abundance between HCC and LC. Only features with LDA score (log 10) > 2.0 and P < 0.05 are shown.


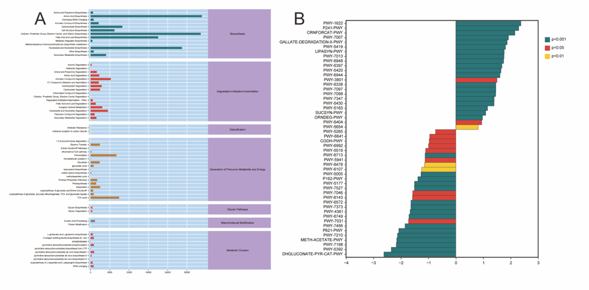


**Supplementary Figure 3**. (**A)**. Representative pathways from MetaCyc database between the HCC and LC groups. (**B)**. Significant biological pathways between the two groups based on MetaCyc database.


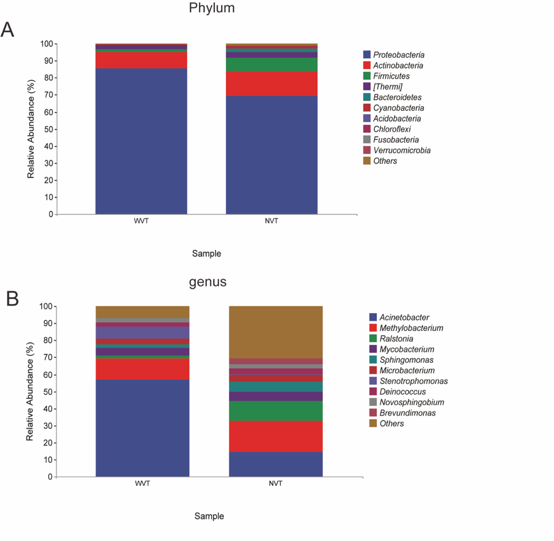


**Supplementary Figure 4.** The relative abundance of microbial taxa at **﻿(A)** phylum and **(B)** genus levels; phyla or genera with a relative abundance <1% in each sample are merged into others.


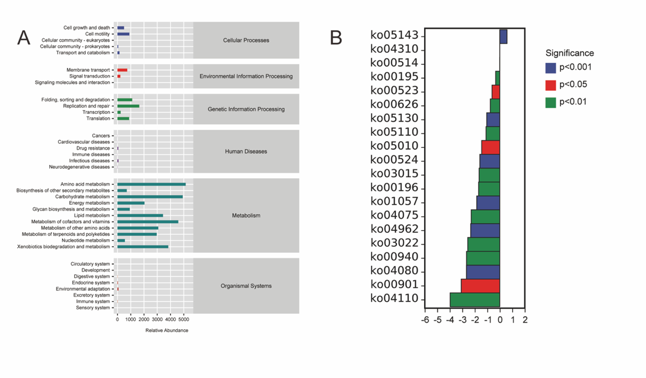


**Supplementary Figure 5.** (**A)**. Representative pathways from KEEG database between the WVT and NVT groups. (**B)**. Significant biological pathways between the two groups based on KEEG database.


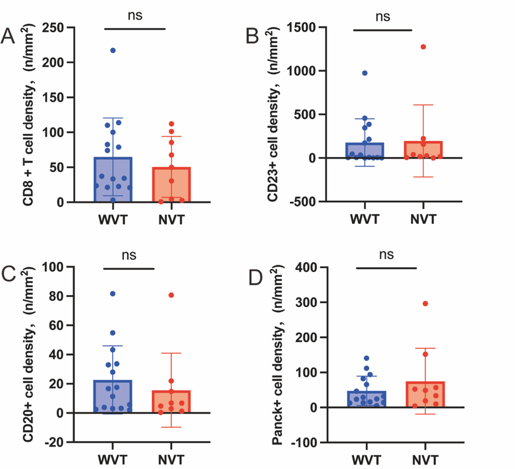


**Supplementary Figure 6.** Quantitative analysis of four immune cells in the WVT and NVT group. The density of **(A)** CD8 + T cells, **(B)** CD23+ cells, **(C)** CD20+ cells, and **(D)** PANCK+ cells in the tissue of WVT and NVT groups.
